# A Case Report of Statin-Induced Immune-Mediated Necrotizing Myopathy Treatment Challenges

**DOI:** 10.1155/2022/4647227

**Published:** 2022-05-31

**Authors:** Anwar I. Joudeh, Mhd Kutaiba Albuni, Sara Seife Hassen, Phool Iqbal, Elsaid Mohamed Aziz Bedair, Salah Mahdi

**Affiliations:** ^1^Department of Internal Medicine, Al-Khor Hospital, Hamad Medical Corporation, Doha, Postcode 3050, Qatar; ^2^Department of Internal Medicine, Hamad General Hospital, Hamad Medical Corporation, Doha, Postcode 3050, Qatar; ^3^Department of Critical Care, Hamad General Hospital, Hamad Medical Corporation, Doha, Postcode 3050, Qatar; ^4^Department of Radiology, Al-Khor Hospital, Hamad Medical Corporation, Doha, Postcode 3050, Qatar

## Abstract

Statin-induced necrotizing autoimmune myopathy is an immune-mediated necrotizing myopathy related to the use of statins. It is a very rare disease, which usually presents with proximal muscle weakness and frank elevation in creatine kinase levels. Stopping statin and the use of immunosuppressive therapy are considered the mainstay therapy. Use of steroids in patients with inflammatory myopathy can be complicated by steroid-induced myopathy. Herein, we present a case of a 55-year-old patient with statin-induced necrotizing autoimmune myopathy based on the presence of proximal muscle weakness, magnetic resonance findings, suggestive muscle biopsy features, and positive anti-HMGCR autoantibodies. The patient was treated with triple immunosuppressive therapy with a particularly good response to intravenous immunoglobulin. This report highlights the importance of timely diagnosis and early use of combined immunosuppressive therapy to improve patients' outcome affected by this rare disease.

## 1. Introduction

Statins are among the most commonly prescribed medications for primary and secondary prevention of cardiovascular disease in eligible patients. Statin-associated muscle symptoms represent a wide spectrum of clinical manifestations ranging from nonspecific muscle aches to severe necrotizing myositis [1]. Statin-induced immune-mediated necrotizing myopathy (IMNM) is a distinctive disease that persists and sometimes progresses after withdrawal of the statin [2]. The exact pathophysiological mechanism of this condition is not fully understood, but it is postulated that development of anti-3-hydroxy-3-methylglutaryl-coenzyme A reductase (anti-HMGCR) autoantibodies is linked to statin exposure in genetically susceptible patients particularly those with class II major histocompatibility complex (MHC) allele DRB1^*∗*^11 : 01 [3]. In vivo studies also supported the pathogenic effect of anti-HMGCR antibodies on myofiber injury through a complement-mediated mechanism which makes complement-targeting therapies and plasma exchange plausible treatment options [4]. Herein, we describe treatment challenges of a 55-year-old man who developed statin-induced IMNM 30 months after starting atorvastatin.

## 2. Case Presentation

A 55-year-old male patient with a history of type 2 diabetes mellitus, hypertension, and dyslipidemia presented to the family clinic for a periodic review. He was noticed to have high liver enzymes; therefore, the general practitioner stopped atorvastatin 20 mg which was originally prescribed two and a half year ago. Upon further questioning, he reported a history of fatigability with a 7-kilogram weight loss over the past 1 year. Ultrasound abdomen done at that time was only remarkable for mild fatty infiltration of the liver, and his hepatitis profile was negative. Five months later, the patient presented to the emergency department complaining of progressive muscle weakness with pain and a difficulty in standing up from the sitting position and holding his arms above the head. He had no anorexia, difficulty of swallowing, diplopia, numbness, or lower limb claudication. He had no skin rash, joint pain, or morning stiffness, and there was no family history of malignancy, autoimmune diseases, or neuromuscular disorders. His current medications included only metformin and lisinopril.

On initial assessment, his vital signs were within the normal range. Musculoskeletal examination revealed proximal muscle weakness that is greater in the lower extremities (upper limb power 4/5 proximally and 5/5 distally, lower limb power 2/5 proximally and 4/5 distally). He had normal muscle tone, reflexes, and sensory examination. The rest of systemic examination was also noncontributory. Laboratory investigations revealed a significant rise in creatinine kinase (CK) 11029 IU, aspartate aminotransferase (AST) 275 U/L, alanine aminotransferase (ALT) 365 U/L, lactic dehydrogenase (LDH) 799 U/L, and myoglobin 1822 ng/ml. However, gamma-glutamyltransferase (GGT), alkaline phosphatase (ALP), thyroid-stimulating hormone, renal functions, serum electrolytes, haemoglobin A1C, full blood count, and vitamin D and B12 levels were all within the normal range.

**Figure 1:**
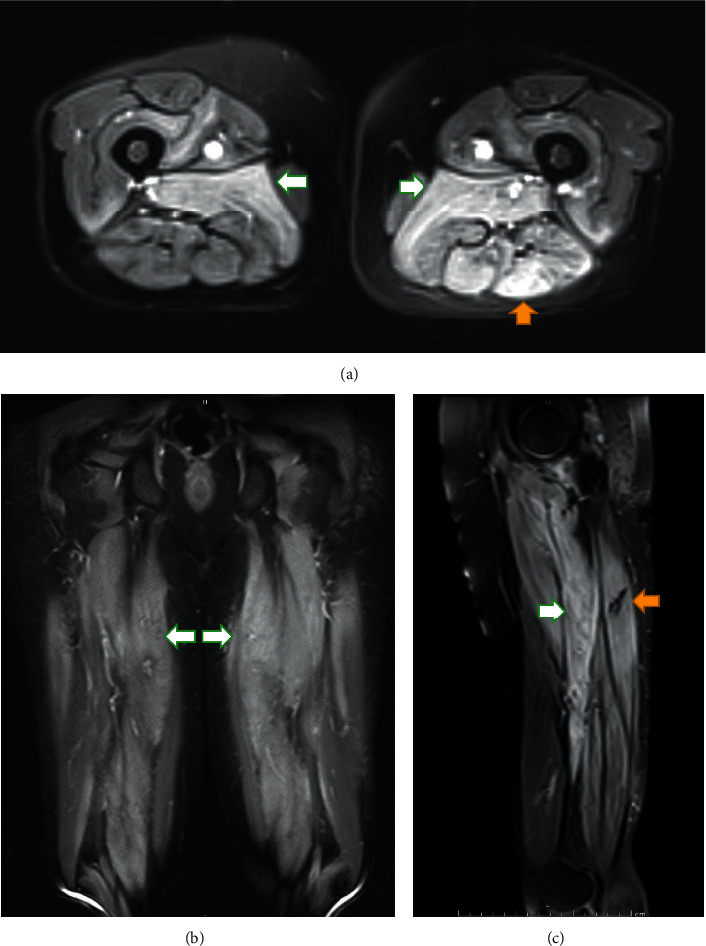
MRI of both thighs ((a) T2 tse fat sat axial, (b) T2-tirm-fs coronal, and (c) T2 tse-fs sagittal left thigh) showing a considerable degree of slightly heterogenous relative hyperintensity of the adductor muscles of both thighs (white arrows) as well as the hamstring muscles of the left thigh (yellow arrows) with no gross increased volume of the muscles appearance. These findings raise the possibility of partial atrophy and intrasubstance edema possibly due to the effects of chronic myositis/myopathy with secondary early muscle atrophic changes.

Computed tomography of the chest, abdomen, and pelvis was performed to rule out any associated occult malignancy, and it was unremarkable. Magnetic resonance imaging of both thighs showed diffuse signal changes exhibiting hyperintense signal on T2 and STIR sequences involving bilateral adductor groups and posterior compartment muscles of the thighs giving the picture of myositis (Figure 1).

Electromyography (EMG) study reported irritable myopathy with severe involvement of the proximal muscles suggestive of immune-mediated necrotizing myopathy. Subsequently, a muscle biopsy was performed which showed rare necrotic fibers without regenerating fibers or inflammatory changes. We checked anti-HMGCR antibodies' level which came back positive with a value of 208.1 CU (normal: less than 20). The rest of autoimmune workup for myositis was negative including anti-SSA 52KD antibody, ANA, anti-Jo-1, anti-La, anti-Scl70, anti-U1 RNP, anti-U2 RNP, anti-U3 RNP, anti-PL-12, anti-PL-7, anti-OJ, anti-EJ, anti-SRP, anti-Ku, anti-PM/SCL100, anti-Mi-2, anti-TIF-1, anti-MDA5, and anti-NXP-2 antibodies.

Based on the above discussion, the patient was diagnosed to have statin-induced IMNM and was started on pulse steroid therapy with intravenous methylprednisolone at 1000 mg daily for 3 days followed by prednisolone 60 mg daily together with intravenous immunoglobulin (IVIG) at 0.4 gm/kg daily for 5 days. He showed significant improvement of muscle strength, and CK dropped from 14866 U/L on the day of starting immunosuppressive therapy to 4800 U/L five days later. With the help of intensive physiotherapy, he was able to ambulate without assistance and was discharged home on oral prednisolone 40 mg daily in a tapering dose.

Despite the initial improvement in this patient clinical status, he presented 3 weeks after discharge from hospital with worsening muscle weakness without increase in muscle enzymes (CK = 2350 U/L). Upon further inquiry, the patient stated that he used a double dose of oral steroids without tapering by mistake. In the assumption of possible steroid-induced myopathy, he was admitted again for gradual tapering of steroids and introduction of methotrexate as a steroid-sparing agent. His blood glucose readings remained within the acceptable range during his readmission, and he was commenced on another cycle of IVIG for 5 days together with intensive physiotherapy. Thereafter, the patient was transferred to a long-term rehabilitation facility for around four weeks and was maintained on prednisolone 10 mg daily, methotrexate 20 mg weekly, folic acid 5 mg once weekly, and monthly IVIG at 1 gm/kg.

During the subsequent six months, the patient had recurrent relapses of muscle weakness alongside increase in muscle enzymes each time prednisolone dose was tapered to less than 10 mg daily despite keeping him on monthly IVIG and a weekly dose of methotrexate at 20 mg orally. Therefore, 1 gm of intravenous rituximab infusion was given after counseling the patient about its associated pros and cons, and a repeated dose was given two weeks later. Nevertheless, the patient was still requiring monthly infusion of IVIG for relapsing muscle weakness on the subsequent six months of rituximab use.

## 3. Discussion

Statin-induced IMNM is a rare side effect of statin intake that complicates their use in two to three patients for every 100,000 individuals exposed to statin [5]. It is considered as a pure autoimmune myopathy that does not improve after withdrawal of the medication and responds to immunosuppressive therapy [6]. Patients with statin-induced IMNM usually have progressive proximal muscle weakness, high serum muscle enzyme levels (CK levels ranging between 2,000 and 20,000 IU/L), myopathic features on electromyography, and muscle fiber necrosis and regeneration with scarce inflammation [7].

Unlike self-limited forms of statin myotoxicity, the pharmacological properties of statins (being hydrophilic or lipophilic, dose of statin used) do not seem to be associated with the development of statin-induced IMNM. However, atorvastatin, simvastatin, and pravastatin were linked to confirmed cases of statin-induced IMNM although this association could be linked to prescribing practice and do not necessarily indicate a causal relationship [8–10]. In a case series by Grable‐Esposito et al. on twenty-five patients with statin-induced IMNM, the average duration of treatment with statin prior to onset of symptoms was three years with a range of two months to ten years [9]. The patient in our case was diagnosed with this condition 30 months after using atorvastatin although he started to develop symptoms around one year prior to diagnosis.

Identification of anti-HMGCR antibodies supports the diagnosis of statin-induced IMNM in the appropriate clinical settings with a high sensitivity and specificity (94.4% and 99.3%, respectively) [6, 11]. Nevertheless, not all patients with positive anti-HMGCR antibodies were exposed to statins [12]. According to Mammen et al., around one-third of patients with anti-HMGCR-positive IMNM were not exposed to statin before and those patients were characteristically younger with higher levels of inflammation [12]. In fact, IMNM is considered a heterogenous disease. In 2016, the European Neuromuscular Center categorized IMNM into three subtypes: anti-signal recognition particle (anti-SRP) myopathy, anti-HMGCR myopathy (statin-exposed and nonstatin exposed), and antibody-negative IMNM. Although these subtypes share many common clinicopathological features, they are distinct categories with variable degrees of muscle involvement severity, extramuscular involvement, and response to individual therapies [13].

Werner et al. studied the association of anti-HMGCR antibodies with disease activity and serum muscle enzyme level in 55 patients with anti-HMGCR-positive IMNM (including 40 patients exposed to statin). The authors found that the initial levels of anti-HMGCR antibodies correlated with serum muscle enzyme levels and muscle weakness in all patients, and they found declining antibody titers and improved muscle strength with immunosuppressive therapy. However, statistically significant improvement was only noticed in statin-induced anti-HMGCR-positive IMNM suggesting a phenotypically different disease between statin-induced and statin-unexposed anti-HMGCR-positive patients [14].

Till date, there are no clinical trials to guide management of statin-induced IMNM. Most treatment recommendations were mainly based on observational evidence, case series, and previous experience of other inflammatory myopathies [7, 15]. However, early identification, stopping statin, and a combination of immunosuppressive therapy are considered the mainstay of treatment [16]. Several immunosuppressive agents were used including corticosteroids, methotrexate, azathioprine, calcineurin antagonists and mycophenolate mofetil, intravenous immunoglobulin, and rituximab [13]. Most cases described in the literature were treated with multiple immunosuppressive agents to control the disease activity including two oral agents plus IVIG [13, 14, 17]. Expert opinions suggested that intravenous immunoglobulin could be particularly useful in patients with diabetes mellitus [5, 18]. However, glucocorticoids alone are not usually adequate and should be used in combination with another immunomodulatory agent. Similarly, IVIG monotherapy is not always effective, and additional immunosuppressive agents are frequently required for maintenance therapy [13, 14, 17]. In this case report, the patient was improving clinically and biochemically each time he received a dose of IVIG but he frequently relapsed despite the use of rituximab, methotrexate, and a low dose of oral steroids.

Management of this patient was further complicated by steroid-induced myopathy which is a clinical diagnosis that requires a high index of suspicion. Steroid-induced myopathy in patients with inflammatory myopathy should be suspected when patients develop worsening muscle weakness accompanied by declining or static creatine kinase levels. Clinical improvement three to four weeks after the discontinuation of steroids can also be supportive [19]. Higher doses of steroids are usually associated with a higher risk of developing myopathy which could present in an acute or a chronic form [19]. In our case, the patient took a double dose of oral steroid (40–80 mg), and his laboratory investigations showed downtrending CK levels with normal inflammatory markers and serum electrolyte levels. This highlights the importance of comprehensive clinical assessment in evaluating patients with complex medical conditions as in this case.

### 3.1. Patient Perspective

The patient has kindly shared his insights and thoughts on the management and impact of this disease on his life. He believes that maintaining effective and regular patient-physician communication was the main factor that helped him to get through this disease process. He thinks that it was extremely difficult for him to become dependent on his family who took good care of him, but that deeply affected his morals and he developed clinical depression for more than one year. Regarding statin use, patients stated that he did not receive enough education on this medicine at the time of starting this treatment nor at the subsequent follow-up visits in primary care clinics. He believes that family medicine physicians should be more vigilant in providing education to patients and in recognizing statin-associated muscle symptoms.

## 4. Conclusion

In summary, we presented a case of statin-induced IMNM diagnosis and treatment challenges. Although this disease is a rare side effect of statin use, physicians should maintain high index of suspicion for this rare entity in patients with statin-associated muscle symptoms who do not improve after stopping the medication. Prompt diagnosis and early use of combination of immunosuppressive therapy cannot be overrated. As the knowledge is rapidly accumulating, it is important to identify particular types of statin associated with this potentially serious side effect and build prediction tools to avoid their use in high-risk patients. Finally, patient education is the cornerstone of successful management in patients with chronic diseases.

## Data Availability

Data can be obtained from the corresponding author upon request.

## Supplementary Materials

Supplementary Materials The supplementary file contains the CARE checklist which we followed in reporting this case report (available at https://www.care-statement.org/checklist).
