# Supplementary material for: A Case Report of Statin-Induced Immune-Mediated Necrotizing Myopathy Treatment Challenges
Source: Case Rep Rheumatol. 2022 May 31;2022:4647227. doi: 10.1155/2022/4647227 (PMC9173901; doi:10.1155/2022/4647227)
Supplement: Supplementary Materials — The supplementary file contains the CARE checklist which we followed in reporting this case report (available at https://www.care-statement.org/checklist). [file 4647227.f1.docx]

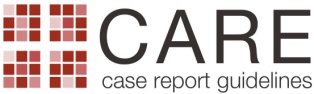
CARE Checklist of information to include when writing a case report
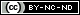


**Topic Item Checklist item description Reported on Line**

**Title 1** The diagnosis or intervention of primary focus followed by the words “case report” P1

**Key Words 2** 2 to 5 key words that identify diagnoses or interventions in this case report, including "case report" P2

Abstract

**(no references)**

**3a** Introduction: What is unique about this case and what does it add to the scientific literature? P2

**3b** Main symptoms and/or important clinical findings P2

**3c** The main diagnoses, therapeutic interventions, and outcomes P2

**3d** Conclusion—What is the main “take-away” lesson(s) from this case? P2

**Introduction 4** One or two paragraphs summarizing why this case is unique (**may include references**) P3

**Patient Information 5a** De-identified patient specific information P3

**5b** Primary concerns and symptoms of the patient P3

**5c** Medical, family, and psycho-social history including relevant genetic information NA

**5d** Relevant past interventions with outcomes P3

Clinical Findings

**Timeline**

**Diagnostic Assessment**

**Therapeutic Intervention**

**Follow-up and Outcomes**

1. Describe significant physical examination (PE) and important clinical findings P4
2. Historical and current information from this episode of care organized as a timeline P5

**8a** Diagnostic testing (such as PE, laboratory testing, imaging, surveys). P5

**8b** Diagnostic challenges (such as access to testing, financial, or cultural) P5

**8c** Diagnosis (including other diagnoses considered) P5

**8d** Prognosis (such as staging in oncology) where applicable NA

**9a** Types of therapeutic intervention (such as pharmacologic, surgical, preventive, self-care) P5

**9b** Administration of therapeutic intervention (such as dosage, strength, duration) P5

**9c** Changes in therapeutic intervention (with rationale) P5

**10a** Clinician and patient-assessed outcomes (if available) P5

**10b** Important follow-up diagnostic and other test results P5

**10c** Intervention adherence and tolerability (How was this assessed?) P5

**10d** Adverse and unanticipated events P5

**Discussion 11a** A scientific discussion of the strengths AND limitations associated with this case report P6-8

**11b** Discussion of the relevant medical literature **with references** P6-8

**11c** The scientific rationale for any conclusions (including assessment of possible causes) P6-8

**11d** The primary “take-away” lessons of this case report (without references) in a one paragraph conclusion P8

**Patient Perspective 12** The patient should share their perspective in one to two paragraphs on the treatment(s) they received P8

**Informed Consent 13** Did the patient give informed consent? Please provide if requested . . . . . . . . . . . . . . . . . . . . . . . . . . . . . . . . . . . . . . **Yes No**
